# TNF-Receptor-Associated Factor 3 in *Litopenaeus vannamei* Restricts White Spot Syndrome Virus Infection Through the IRF-Vago Antiviral Pathway

**DOI:** 10.3389/fimmu.2020.02110

**Published:** 2020-09-11

**Authors:** Haoyang Li, Qihui Fu, Sheng Wang, Rongjian Chen, Xiewu Jiang, Peng Zhu, Jianguo He, Chaozheng Li

**Affiliations:** ^1^Southern Marine Science and Engineering Guangdong Laboratory (Zhuhai)/State Key Laboratory of Biocontrol, School of Marine Sciences, Sun Yat-sen University, Guangzhou, China; ^2^Guangdong Hisenor Group Co., Ltd., Guangzhou, China; ^3^Guangxi Key Laboratory of Beibu Gulf Marine Biodiversity Conservation, Beibu Gluf University, Qinzhou, China

**Keywords:** *Litopenaeus vannamei*, TRAF3, NF-κB, IRF, WSSV

## Abstract

Tumor necrosis factor receptor (TNFR)-associated factors (TRAFs) are vital signaling adaptor proteins for the innate immune response and are involved in many important pathways, such as the NF-κB- and interferon regulatory factor (IRF)-activated signaling pathways. In this study, the TRAF3 ortholog from the shrimp *Litopenaeus vannamei* (LvTRAF3) was cloned and characterized. LvTRAF3 has a transcript of 3,865 bp, with an open reading frame (ORF) of 1,002 bp and encodes a polypeptide of 333 amino acids, including a conserved TRAF-C domain. The expression of LvTRAF3 in the intestine and hemocyte was up-regulated in response to poly (I:C) challenge and white spot syndrome virus (WSSV) infection. RNAi knockdown of LvTRAF3 *in vivo* significantly increased WSSV gene transcription, viral loads, and mortality in WSSV-infected shrimp. Next, we found that LvTRAF3 was not able to induce the activation of the NF-κB pathway, which was crucial for synthesis of antimicrobial peptides (AMPs), which mediate antiviral immunity. Specifically, in dual-luciferase reporter assays, LvTRAF3 could not activate several types of promoters with NF-κB binding sites, including those from WSSV genes (*wsv069*, *wsv056*, and *wsv403*), *Drosophila* AMPs or shrimp AMPs. Accordingly, the mRNA levels of shrimp AMPs did not significantly change when TRAF3 was knocked down during WSSV infection. Instead, we found that LvTRAF3 signaled through the IRF-Vago antiviral cascade. LvTRAF3 functioned upstream of LvIRF to regulate the expression of *LvVago4* and *LvVago5* during WSSV infection *in vivo*. Taken together, these data provide experimental evidence of the participation of LvTRAF3 in the host defense to WSSV through the activation of the IRF-Vago pathway but not the NF-κB pathway.

## Introduction

Tumor necrosis factor receptor (TNFR)-associated factors (TRAFs) are intracellular signal transducers for a number of members of the immune receptor superfamily, which converge on inducing the production of proinflammatory factors, interferons (IFNs) and/or antimicrobial peptides (AMPs) (1). A total of six TRAF families, TRAF1–6, have been identified in mammals, most of which are implicated with several innate immune responses (1). Upon pathogenic infection, a TRAF6 forms a signal transduction complex with myeloid differentiation factor 88 (MyD88), IL-1 receptor-associated kinase-4 (IRAK4) and IRAK1 to activate the downstream NF-κB pathway and trigger the expression of immune-related effectors (2–4). Conversely, TRAF3 plays a negative regulatory role in the MyD88-dependent Toll like receptor (TLR) pathway through inhibiting the formation of the complexes for IRAK1, IRAK4, and TRAF6 (5). In addition to the NF-κB pathway, TRAF family members are involved in signaling regulation in the interferon regulatory factor (IRF)-IFN pathway. In mammals, TRAF2, TRAF3, and TRAF6 can bind with TRIF, the adaptor protein of the TLR3/4 pathway, to regulate the activation of IRF3 and finally promote the expression of type I IFNs (5, 6). In the RIG-I-like receptor (RLR) pathway, the activated mitochondrial antiviral-signaling protein (MAVS) can recruit TRAF2, TRAF3, TRAF5, and/or TRAF6, then these TRAFs are ubiquitinated, followed by the recruitment of the TANK/IKKε/TBK1 complex, and finally IRF3 is recruited and phosphorylated (7). In addition, TRAF3 is involved in the STING-mediated activation of IRF3 (8). In general, most TRAF family members participate in the regulation of the NF-κB and IRF-mediated innate immune pathways.

In *Drosophila melanogaster*, three TRAFs, named dTRAF4 (also called dTRAF1), dTRAF6 (also called dTRAF2) and dTRAF3, have been identified. dTRAF4 is homologous to mammalian TRAF4, and has a conserved TRAF-C domain and seven zinc fingers (9). dTRAF4 was found to regulate JNK pathway activation and participate in *Drosophila* embryo development and differentiation (10). dTRAF6 is homologous to mammalian TRAF6 and has similar functions in inducing the activation of the NF-κB pathway (9). Interestingly, dTRAF3 is likely to have derived from a common precursor to the mammalian TRAF1, 2, 3, and 5 genes (9). A recent study showed that viral infection can enhance lipolysis through the TRAF3-AMPK/WTS-Atg1 pathway to increase intestinal resistance (11). In all, the regulatory relationship between invertebrate TRAFs and classical innate immune signaling pathways remains unclear.

The shrimp species *Litopenaeus vannamei* has become one of the most important cultured species in the world. With high-density tolerance and a rapid growth rate, *L. vannamei* production accounts for 75% of the world’s shrimp production each year (12). Recently, several kinds of shrimp diseases have threatened shrimp aquaculture, especially the white spot syndrome (WSS) caused by white spot syndrome virus (WSSV) (13). Within 3–10 days of infection, the mortality rate of WSSV-infected shrimp can reach up to 100%, which has led to serious economic losses in shrimp cultures (14). In addition, to prevent and control the diseases of cultured shrimp, the selection and breeding of shrimp with strong disease resistance have become an important measure for fundamentally improving the disease resistance of shrimp. Both shrimp disease prevention and genetic improvement are based on theoretical support from the study of immune genes. In shrimp, the IRF-Vago and NF-κB pathways have been demonstrated to be crucial for antiviral immunity (12), but the mechanism underlying their signal transduction in WSSV infection is still poorly understood.

In this study, we cloned a new TRAF3 ortholog from *L. vannamei* and explored its function during WSSV infection. We found that LvTRAF3 could signal through the IRF-Vago pathway, but not the NF-κB pathway, to confer protective immunity for shrimp from viral infection. These results provide some insights into the antiviral function of invertebrate TRAF3 members.

## Materials and Methods

### Cloning of Full Length of LvTRAF3 cDNA

An expressed sequence tag (EST) encoding a putative TRAF3 protein was retrieved from *L. vannamei* transcriptome data to obtain the 3′- and 5′-ends of LvTRAF3 using gene specific primers via the rapid amplification of cDNA ends (RACE) method as previously described (Table 1) (15). The cDNA template for RACE-PCR was prepared with the SMARTer PCR cDNA Synthesis Kit (Clontech, Japan). The first-round PCR amplifications were conducted on 10-fold dilutions of SMARTer RACE cDNA with either Universal Primer A Mix (UPM)/LvTRAF3-5RACE1 (for 5′-RACE) or UPM/LvTRAF3-3RACE1 (for 3′-RACE), respectively. The products from the first-round PCR were diluted 50-fold as templates for a second round of PCR. Primers of Nested Universal Primer A (NUP) and LvTRAF3-5RACE2 or 3RACE2 were used for a second round of 5′- and 3′-RACE PCR, respectively. The final products were cloned into the pMD-20T Cloning Vector (TaKaRa, Japan) and 12 positive clones were selected and sequenced.

**TABLE 1:** Summary of primers in this study.

**RACE**	
LvTRAF3-5RACE1	CTGGGCAATGGCTAGGAGATCCGGT
LvTRAF3-5RACE2	ACTCAATCTCCTCGTTGCCCTGCTG
LvTRAF3-3RACE1	GTTGTCACACTAACTTTGTCTTTTG
LvTRAF3-3RACE2	TTCATAGGGTGCAATACCTTGGCTT
**Protein expression**	
LvTRAF3-F	GGGGTACCATGGAGCGAGCGGTGCTGTTGTGTG
LvTRAF3-R	TTGGGCCCTTAGTAGTAGGTGAAGACCCTCCAGCCG
LvDorsal-F	AGGGGTACCATGTTTGTTGCCCAGCGTACTTCC
LvDorsal-R	AACGGGCCCTCACATATCAGAAAATATCCAAAAC
LvRelish-F	AGGGGTACCATGGTGAGAGGTGACAGAGGTGG
LvRelish-R	AACGGGCCCTCACGCCTGGTCCAGTACAGCTACA CATTCC
DmDorsal-F	CGGGGTACCATGTTTCCGAACCAGAACAATGGAGCCG
DmDorsal-R	TGCTCTAGATTACGTGGATATGGACAGGTTCGATATCT
DmRelish-F	CCGGAATTCATGAACATGAATCAGTACTACGACC
DmRelish-R	TGCTCTAGATTATCAAGTTGGGTTAACCAGTAGGG
**qRT-PCR**	
LvTRAF3-F	CTCCTAGCCATTGCCCAGAG
LvTRAF3-R	GGTCCACCACCTGTTTCTGC
LvEF-1α-F	TATGCTCCTTTTGGACGTTTTGC
LvEF-1α-R	CCTTTTCTGCGGCCTTGGTAG
VP28-F	AACACCTCCTCCTTCACCC
VP28-R	GGTCTCAGTGCCAGAGTAGGT
wsv056-F	TCTGGCAAGGAGATTATGGAGAACCG
wsv056-R	TTTCTTCGTATTTTCTTCATTGTTGGAGGG
wsv069-F	ACAACAACAGACCCTACCCGCCCCA
wsv069-R	GTTGCTGATAAACTCTTGAAGGAAT
wsv249-F	CCCGGACGGAGACGTGATAA
wsv249-R	ATGATGATGGGCCTTTCTTCTCT
wsv403-F	GGGTGGTTGCTTCAACTCCGT
wsv403-R	TCGGTATAGGTTTGGTGTACGTCTCA
LvLYZ1-F	TACGCGACCGATTACTGGCTAC
LvLYZ1-R	AGTCTTTGCTGCGACCACATTC
LvALF1-F	TTACTTCAATGGCAGGATGTGG
LvALF1-R	GTCCTCCGTGATGAGATTACTCTG
LvCTL3-F	ATGTTCTTCGTGCTCCTGCTGT
LvCTL3-R	GCAGTGGTCGTAAATGTTGTG
LvCTL4-F	GCTTTTACTTCCATCAAGACCAG
LvCTL4-R	TGTTAGGATGTACTCATAAAATTCCCT
LvVago4-F	ACGACGAGTTCACGAATTGGATC
LvVago4-R	ACGGCATCTTACCTCAAGAGTC
LvVago5-F	CTCCATAGCCAGGCACGAAAG
LvVago5-R	GTCAGCACAAGCAGCATCACA
**Absolute qPCR**	
WSSV32678-F	TGTTTTCTGTATGTAATGCGTGTAGGT
WSSV32753-R	CCCACTCCATGGCCTTCA
TaqMan probe-	CAAGTACCCAGGCCCAGTGTCATACGTT
WSSV32706	
**dsRNA templates amplification**	
LvTRAF3-F	CTCAGGACCCAAGACAAGC
LvTRAF3-R	GTCAGCCCGACGTGAATAT
LvTRAF3-T7-F	GGATCCTAATACGACTCACTATAGGCTCAGGAC CCAAGACAAGC
LvTRAF3-T7-R	GGATCCTAATACGACTCACTATAGGGTCAGC CCGACGTGAATAT
GFP-F	CGACGTAAACGGCCACAAGTT
GFP-R	ATGGGGGTGTTCTGCTGGTAG
GFP-T7-F	GGATCCTAATACGACTCACTATAGGCGACGTAAA CGGCCACAAGTT
GFP-T7-R	GGATCCTAATACGACTCACTATAGGATGGGGGTG TTCTGCTGGTAG
LvIRF-F	ATGCCGCCATCTTTCACCAATG
LvIRF-R	CTACGGCAACGTCCTCTCGCCGGCA
LvIRF-T7-F	GGATCCTAATACGACTCACTATAGGATGCCGCCAT CTTTCACCAATG
LvIRF-T7-R	GGATCCTAATACGACTCACTATAGGCTACGGCAACGTC CTCTCGCCGGCA

### Sequence and Phylogenetic Analysis of LvTRAF3

Protein domains for LvTRAF3 were predicted using the SMART program^1^ (16). Protein sequences of TRAFs from other species were found using the National Center for Biotechnology Information (NCBI^2^) database. Sequences of LvTRAF3 and TRAF3 homologs from other species were aligned using Clustal X v 2.0 (17) and visualized using GeneDoc software^3^ (18). Phylogenetic trees were constructed via MEGA 5.0 with the neighbor joining (NJ) method (19).

### Plasmid Construction

A GFP coding sequence was synthesized and cloned into pAc5.1/V5-His A (Invitrogen) using *Bst*BI/*Pme*I sites to replace the V5-His tag, generating pAc5.1A-GFP for GFP-tagged protein expression (15). The open reading frame (ORF) without a stop codon of *LvTRAF3* was then cloned into pAc5.1A-GFP. The ORFs without stop codons of *LvTRAF3*, *LvDorsal* (Accession No. ROT84343.1), *LvRelish* (Accession No. ABR14713.1), *DmDorsal* (Accession No. NP_724052.1), and *DmRelish* (Accession No. NP_477094.1) were cloned into the *Kpn*I/*Apa*I sites of pAc5.1A vector for the expression of V5-tagged proteins, respectively. The 5′ flanking regulatory regions of *Lysozyme 1* (*LYZ1*) (Accession No. ABD65298), *anti-lipopolysaccharide (LPS) factor 1* (*ALF1*) (Accession No. AHG99284.1), *C-type lectin 3* (*CTL3*) (Accession No. AGV68681.1), and *C-type lectin 4* (*CTL4*) (Accession No. AKA64754.1) were cloned into the pGL3-Basic vector (Promega). Primer sequences are listed in Table 1 and Supplementary Table S1.

### Confocal Laser Scanning Microscopy

*Drosophila* S2 cells were seeded onto glass slides in 12-well plates. After being cultured 24 h, S2 cells were then transfected with pAc5.1A-LvTRAF3-GFP or pAc5.1A-GFP (as a control) using the FuGENE HD Transfection Reagent (Promega). After 36 h, cells were fixed with 4% paraformaldehyde. Hochest 33258 (Beyotime, China) was used to stain cells for 5 min, and then the cells were washed three times with PBS, and finally, visualized with confocal laser scanning microscopy (Leica TCS-SP5, Germany).

The hemocytes from double stranded RNAs (dsRNA)-injected shrimp at 48 hpi were centrifuged at 3000 *g* for 10 min at 4°C. The cells were washed twice with PBS and spread onto cover slips in a 24-well plate (Corning, Untied States). After 30 min, remove PBS and fixed cells in 4% paraformaldehyde diluted in PBS at 25°C for 15 min. The cells were then permeabilized with methanol at −20°C for 10 min. After washing slides for three times, the hemocytes were blocked with 3% bovine serum albumin (BSA) (diluted in PBS) for 1 h at 25°C and then incubated with a mixture of primary antibodies (1:100, diluted in blocking reagent) overnight (about 8 h) at 4°C. The primary antibodies used in immunofluorescence (IF) were rabbit anti-LvIRF antibody (20) and mouse anti-β-actin antibody (Sigma-Aldrich, United States). The slides were washed with PBS six times and then incubated with 1:1000 diluted anti-rabbit IgG (H + L), F (ab′)2 fragment Alexa Fluor 488 Conjugate (CST, United States), and anti-mouse IgG(H + L), F (ab′)2 Fragment Alexa Fluor 594 Conjugate (CST, United States) for 1 h at 25°C. The cell nuclei were stained with Hoechst 33258 Solution (Beyotime, China) for 10 min. Finally, the slides were observed with a confocal microscope (Leica, Germany) after washing six times with PBS.

### SDS-PAGE and Western Blotting

Hemocytes of dsRNA-injected shrimps were sampled with each sample collected and pooled from five shrimps. The nuclear and cytoplasmic fractions of hemocytes were extracted according to the protocol of NE-PER Nuclear and Cytoplasmic Extraction Reagents (Thermo Fisher Scientific, United States). Samples were boiled for 5 min, separated on SDS-PAGE gels followed by transfer to polyvinylidene difluoride (PVDF) membranes. After blocking in 5% BSA in TBS with 0.1% Tween-20 (TBS-T) for 1 h, membranes were incubated with anti-LvIRF, anti-HSP90 (Abcam, United States) and anti-Histone H3 (CST, United States) for 15 h at 4°C. After washing in TBS-T, membranes were incubated for 1 h at 25°C with horseradish peroxidase (HRP)-labeled Goat secondary antibody to Goat anti-Rabbit IgG (H + L)-HRP or Goat anti-Mouse IgG (H + L)-HRP. Both primary and secondary antibodies were incubated in TBS-T with 0.5% BSA. Membranes were developed with the enhanced chemiluminescent (ECL) blotting substrate (Thermo Fisher Scientific) and chemiluminescence was detected using the 5200 Chemiluminescence Imaging System (Tanon, China).

### qRT-PCR Analysis of LvTRAF3 Expression

Healthy shrimp were provided by a shrimp farm (Guangdong Hisenor Group) in Maoming, Guangdong province, China. Tissues from the gills, hepatopancreases, hemocytes, and intestines were sampled and pooled from 15 shrimp for assaying tissue expression distribution. For immune stimulation assays, the treated groups were injected with 5 μg of poly (I:C) or WSSV (1 × 10^6^ particles, newly extracted) in 50 μl PBS at the second abdominal segment of each shrimp, and the control group was injected with a PBS solution. The hemocytes and intestines of challenged shrimp were sampled at 0, 4, 8, 12, 24, 36, 48, and 72 h post-injection (hpi), and each sample was collected and pooled from 15 shrimp. Total RNA and qRT-PCR were performed as described previously (21). Expression levels of *LvTRAF3* were calculated using the Livak (2^–ΔΔCT^) method after normalization to *L. vannamei EF-1a* (Accession No. GU136229). Primer sequences are listed in Table 1. Each experiment was repeated at least three times.

### Knockdown of LvTRAF3 Expression by dsRNA-Mediated RNAi

Double stranded RNAs specifically targeting the LvTRAF3 gene, as well as GFP, as a control, were synthesized by *in vitro* transcription as previously described with the gene-specific primers listed in Table 1 (22). The lengths of LvTRAF3 and GFP dsRNA were 422 and 504 bp, respectively. The experimental group was injected with LvTRAF3 dsRNA (2 μg/g shrimp), while the control groups were injected with GFP dsRNA (2 μg/g shrimp) and PBS, respectively. The RNA interference efficiency was measured by qRT-PCR. Briefly, total RNA was extracted from hemocytes sampled from the experimental and control groups (nine shrimp from each group) at 48 h post injection and subsequently reverse transcribed into cDNA as a template for qRT-PCR. *L. vannamei EF*-*1a* was used as an internal control. Primer sequences are listed in Table 1. Each experiment was performed at least three times.

### WSSV Challenge Experiments in LvTRAF3-Knockdown Shrimp

To explore whether LvTRAF3 plays a role in defense against WSSV, healthy shrimp (5 ± 0.5 g) were divided into two groups. The control group received GFP dsRNA injection and the RNAi group received LvTRAF3 dsRNA. Forty-eight hours later, shrimp were injected again with 1 × 10^5^ copies of WSSV particles or 50 μl PBS. Shrimp were kept in culture flasks for about 7 days following infection. The survival number was recorded every 4 h. Differences in the mortality levels between treatments were tested for statistical significance using a Kaplan–Meier plot (log-rank χ^2^ test) in GraphPad Prism software.

A parallel experiment was also performed to monitor WSSV replication in LvTRAF3-knockdown shrimp. Briefly, eight samples of hemocytes or gills (each sample pooled from one shrimp) were collected from each group at 48 h post infection for RNA and DNA extraction. Hemocyte RNA was extracted for detecting RNAi efficiency (*LvTRAF3*), and for measuring the expression changes of both viral and host genes, including WSSV genes (*wsv056*, *wsv069*, *wsv249*, *wsv403*, and *VP28*), shrimp NF-κB-mediated genes (*LvALF1*, *LvLYZ1*, *LvCTL3*, and *LvCTL4*) and shrimp IRF-mediated genes (*LvVago4* and *LvVago5).* Gill DNA was also extracted with the TIANGEN Marine Animals DNA Kit (TIANGEN, China) according to the user’s introduction. The viral loads were measured by absolute quantitative PCR with primers WSSV32678-F/WSSV32753-R and a TaqMan fluorogenic probe (Table 1). The WSSV genome copy numbers in 0.1 μg of shrimp gill DNA were then calculated. Primer sequences are listed in Table 1. Each experiment was performed at least three times.

### Dual-Luciferase Reporter Assays

In this study, *Drosophila* S2 cells were used for dual-luciferase reporter assays. Before plasmid transfection, S2 cells were seeded into 96-well plate (TPP, Switzerland), and LvTRAF3 plasmids were transfected the next day using the FuGENE Transfection Reagent (Promega) according to the manufacturer’s recommendations. For dual-luciferase reporter assays, S2 cells in each well of a 96-well plate were transfected with 0.1 μg of reporter gene plasmids (pGL3-*DmMtk*, pGL3-*DmDef*, pGL3-*DmCecA*, pGL3-*DmDipt*, pGL3-*DmAttA*, pGL3-*DmDrs*, pGL3-*LvALF1*, pGL3-*LvLYZ1*, pGL3-*LvCTL3*, or pGL3-*LvCTL4*), 0.01 μg of pRL-TK renilla luciferase plasmid (as an internal control), and 0.1 μg of expression plasmids (pAc5.1A-LvTRAF3) or the empty pAc5.1/V5-His A plasmid (as control). At 48 h post transfection, the activities of the firefly and renilla luciferases were measured according to manufacturer’s instructions. Each experiment was conducted at least three times.

### WSSV Challenge Experiments in Double LvTRAF3/LvIRF-Knockdown Shrimp

In shrimp, the IRF-Vago cascade is crucial for defense against WSSV infection (20). We try to figure out whether LvTRAF3 regulates the expression of LvVago4/5 through LvIRF during WSSV infection, thus a double gene (LvTRAF3/LvIRF) knockdown was performed. Forty-eight hours after dsRNAs injection, shrimp were injected again with 1 × 10^5^ copies of WSSV particles. Shrimp were then kept in culture flasks for about 7 days following infection. The cumulative mortality was recorded every 4 h. Differences in the mortality levels between treatments were tested for statistical significance using a Kaplan–Meier plot (log-rank χ^2^ test) in GraphPad Prism software.

A parallel experiment was also performed to monitor the RNA interference of dsRNA, viral envelope protein *VP28* transcription and WSSV replication. After 48 h after WSSV infection, the hemocytes and gills were collected for RNA extraction and DNA extraction (9 shrimp for RNA extraction and 12 shrimp for DNA extraction from each group). The RNAi efficiency (*LvTRAF3*) and WSSV genes (*wsv056*, *wsv069*, *wsv249*, *wsv403*, and *VP28*) transcription was measured by qRT-PCR, and WSSV replication detection was performed by absolute quantitative PCR. Primer sequences are listed in Table 1. Each experiment was conducted at least three times.

## Results

### Sequence Analysis of LvTRAF3

**FIGURE 1:**
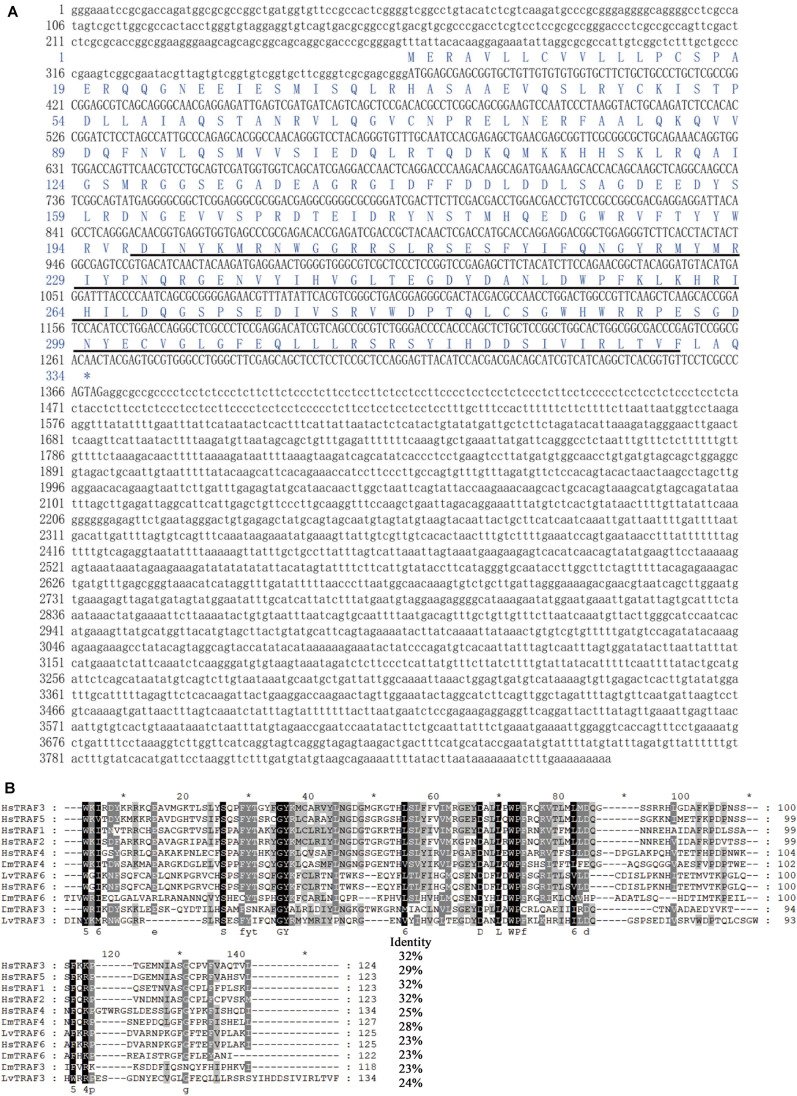
Full-length cDNA and amino acid sequence of LvTRAF3, and multiple sequence alignment with TRAFs. **(A)** The ORF of the nucleotide sequence is shown in upper-case letters, while the 5′ and 3′-UTR sequences are shown in lowercase. Amino acid sequence is represented with one-letter codes above the nucleotide sequence. The TRAF-C domain in the C-terminal is underlined. **(B)** Identical amino acid residues are shaded in black and similar residues are in gray. Amino acid identities of the LvTRAF3 with other TRAFs are shown on the right. Proteins analyzed are listed below: LvTRAF3, *L. vannamei* TRAF3 (Accession No. MN037815); LvTRAF6, *L. vannamei* TRAF6 (113823573); HsTRAF1, *Homo sapiens* TRAF1 (NP_005649.1); HsTRAF2, *H. sapiens* TRAF2 (ADQ89802.1); HsTRAF3, *H. sapiens* TRAF3 (NP_003291.2); HsTRAF4, *H. sapiens* TRAF4 (NP_004286.2); HsTRAF5, *H. sapiens* TRAF5 (CAG38794.1); HsTRAF6, *H. sapiens* TRAF6 (NP_004611.1); DmTRAF4, *D. melanogaster* TRAF4 (NP_477416.1); DmTRAF6, *D. melanogaster* TRAF6 (AAD47895.1); DmTRAF3, *D. melanogaster* TRAF3 (NP_727976.1).

The transcript of *LvTRAF3* was found to be 3,865 bp long, consisting of a 368 bp 5′-untranslated region (UTR), a 2,495 bp 3′-UTR including a poly (A) tail, and a 1,002 bp ORF encoding a polypeptide of 333 amino acids with a calculated molecular weight of 38.6 kDa (Accession No. MN037815). The predicted domain analysis of LvTRAF3 indicated that there was a TRAF-C domain (MATH domain) located at 197–330 amino acids (Figure 1A).

Multiple sequence analysis showed that the TRAF-C domain of *L. vannamei* TRAF3 was similar to that of *Homo sapiens* TRAF3 (32% identity), *H. sapiens* TRAF5 (29% identity), *H. sapiens* TRAF1 (32% identity), *H. sapiens* TRAF2 (32% identity), *H. sapiens* TRAF4 (25% identity), *D. melanogaster* TRAF4 (28% identity), *L. vannamei* TRAF6 (23% identity), *H. sapiens* TRAF6 (23% identity), *D. melanogaster* TRAF6 (23% identity), and *D. melanogaster* TRAF3 (24% identity) (Figure 1B). In general, the TRAF-C domain of LvTRAF3 showed conservation to those of *Drosophila* and human TRAFs, suggesting that LvTRAF3 was a TRAF family member.

**FIGURE 2:**
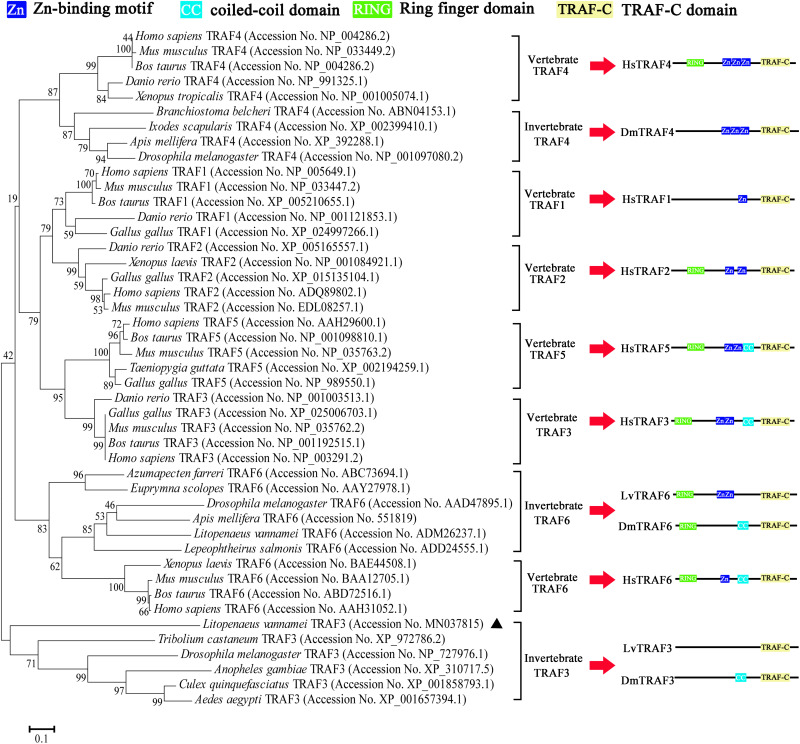
Phylogenetic tree of TRAFs using full-length amino acid sequences from various species. GenBank accession numbers are shown after scientific names of their species. The right side shows the structure of TRAFs belonging to human, *Drosophila*, and shrimp.

According to the NJ phylogenetic tree, the TRAFs from various species could be divided into nine groups, namely vertebrate TRAF4, invertebrate TRAF4, vertebrate TRAF1, vertebrate TRAF2, vertebrate TRAF5, vertebrate TRAF3, invertebrate TRAF6, vertebrate TRAF6, and invertebrate TRAF3. *L. vannamei* TRAF3 (LvTRAF3) was clustered with TRAF3 homologs from invertebrates, including *D. melanogaster*, *Anopheles gambiae*, *Culex quinquefasciatus*, and *Aedes aegypti*, further suggesting that LvTRAF3 was a TRAF3 family member (Figure 2).

### Expression of *LvTRAF3* in Healthy and Immune-Challenged Shrimp

**FIGURE 3:**
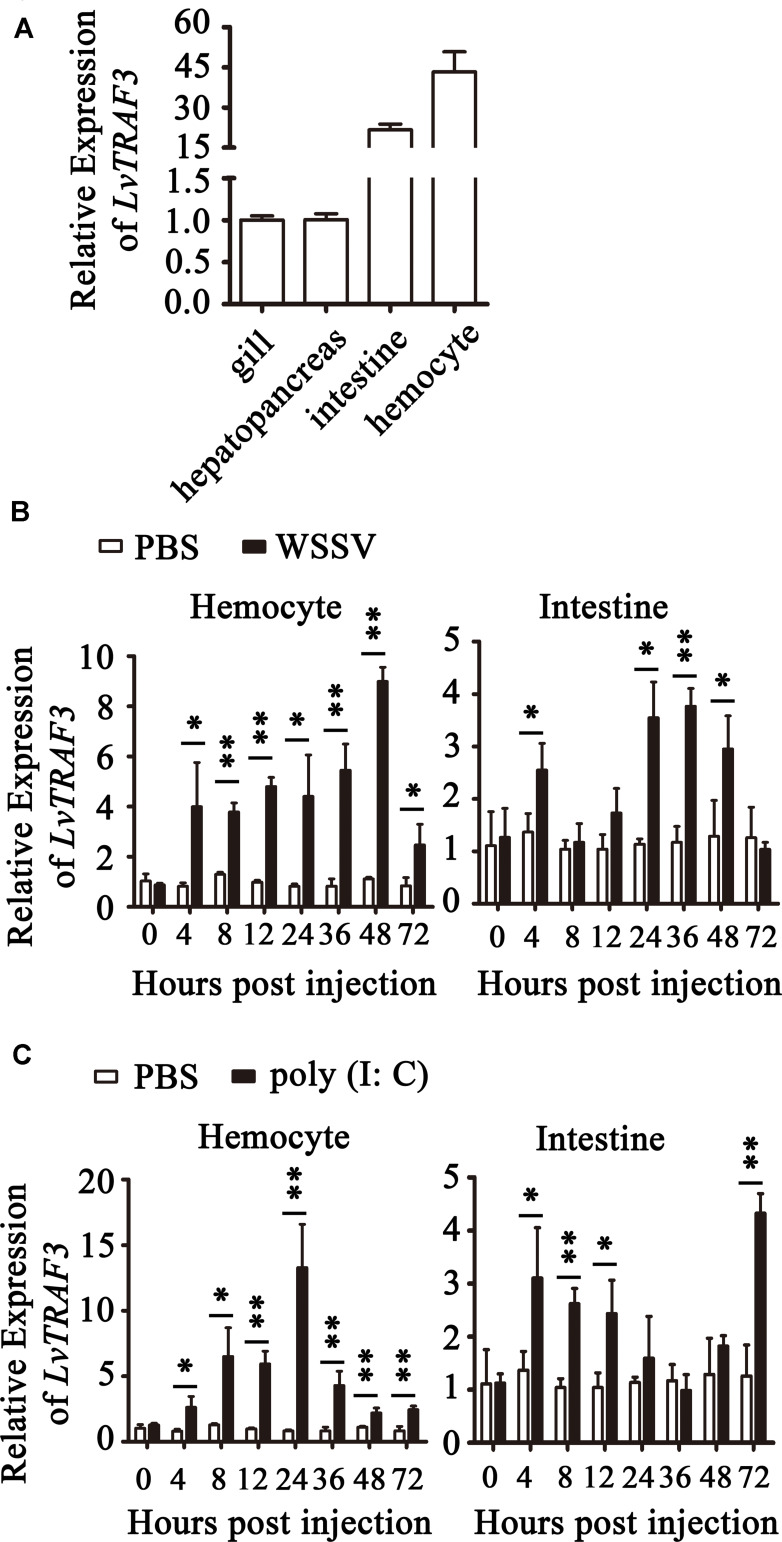
Expression of *LvTRAF3* in healthy and immune challenged shrimp. Expression of *LvTRAF3* was normalized to that of *EF-1*α using the Livak (2^–ΔΔCT^) method and the data is provided as the mean ± SD from triplicate assays. **(A)** Tissue distribution of LvTRAF3 in healthy *L. vannamei*. Expression level in the gill was used as control and set to 1.0. **(B)** Expression profiles of *LvTRAF3* after WSSV infection in hemocytes and the intestine. The expression level at each time point was normalized to PBS-injected group. (^∗^*p* < 0.05, ^∗∗^*p* < 0.01). **(C)** Expression profiles of *LvTRAF3* after poly (I:C) stimulation in hemocyte and intestine. The expression level at each time point was normalized to the PBS-injected group. (^∗^*p* < 0.05, ^∗∗^*p* < 0.01).

Tissue distribution analysis showed that *LvTRAF3* was expressed highly in the intestine and hemocyte, with ∼22-fold and ∼43-fold levels over that in the gill (set to 1.0), respectively (Figure 3A).

The shrimp hemocytes are leukocyte-like blood cells with phagocytic functions, while the intestine is the crucial organ involved in immune defense against bacterial infection (23). In the hemocyte of WSSV-infected shrimp, the expression of *LvTRAF3* was dramatically up-regulated at 4 h with a 3.99-fold increase, and then stayed at a high level (8.99-fold) at 48 h. Infected with WSSV, *LvTRAF3* expression in the intestine was induced to a peak (2.55-fold) at 4 h, remained at a high level during 24–48 h with 3. 55-, 3. 77-, and 2.95-fold increases at 24, 36, and 48 h, respectively (Figure 3B). Then we explored the effect of poly (I:C), a viral nucleic acid mimic, on *LvTRAF3* expression. The expression of *LvTRAF3* in hemocyte was gradually up-regulated from 4 to 72 h, with a 13.29-fold peak at 24 h. In intestine, the injection of poly (I:C) induced *LvTRAF3* transcription from 4 to 12 h, with 3. 11-, 2. 62-, and 2.43-fold increases at 4, 8, and 12 h, respectively, and then reached a peak at 72 h with a 4.33-fold increase. The control group injected with PBS did not show obvious changes in *LvTRAF3* expression (Figure 3C).

### Subcellular Localization of LvTRAF3

**FIGURE 4:**
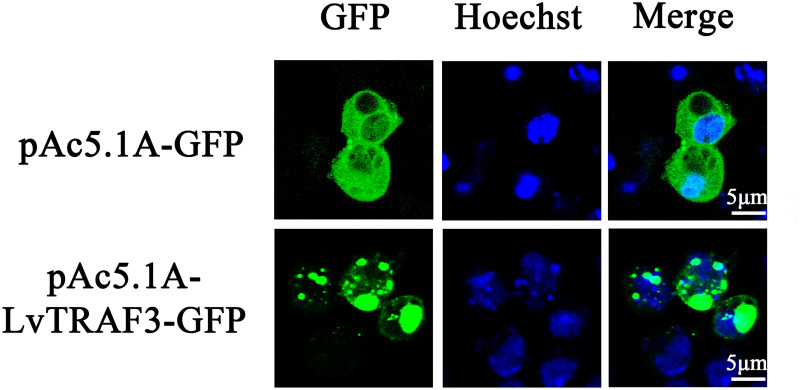
Subcellular localization of LvTRAF3 in *Drosophila* S2 cells. *Drosophila* S2 cells were transfected with plasmids pAc5.1A-LvTRAF3-GFP and pAc5.1A-GFP. At 36 h post-transfection, cells were observed using a Leica laser scanning confocal microscope.

Subcellular localization plays an important role in the study of protein function. Vector pAc5.1A-LvTRAF3-GFP was constructed by inserting the full-length coding sequences of *LvTRAF3* into the pAc5.1A-GFP vector. To investigate the subcellular distribution of LvTRAF3, pAc5.1A-LvTRAF3-GFP plasmids were transfected into *Drosophila* S2 cells, and the GFP-fusion proteins were visualized using confocal laser scanning microscopy. Figure 4 shows that LvTRAF3 aggregated in the cytoplasm and nucleus, while GFP was distributed uniformly.

### Critical Role of LvTRAF3 in Defense Against WSSV

**FIGURE 5:**
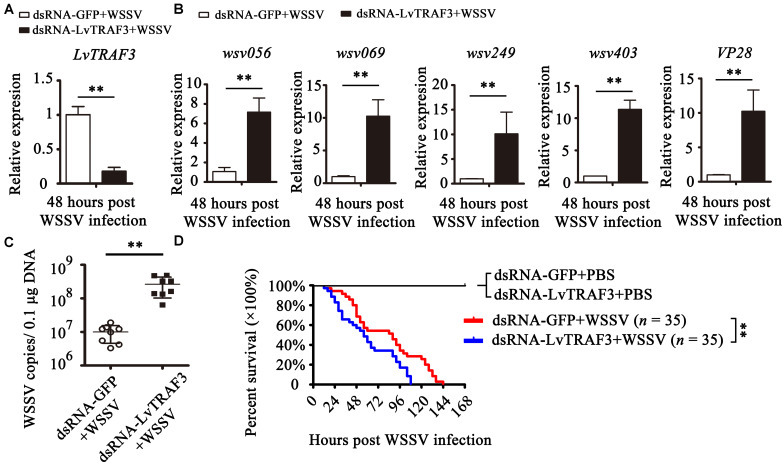
Functional analysis of LvTRAF3 in WSSV infection by RNAi. **(A)** The silencing efficiency of LvTRAF3 was confirmed by qRT-PCR normalized to *EF-1a*. **(B)** Expression levels of *wsv056*, *wsv069*, *wsv249*, *wsv403*, and *VP28* in dsRNA-GFP treated or dsRNA-LvTRAF3 treated shrimp 48 h post WSSV infection. **(C)** Copy number of WSSV in dsRNA-LvTRAF3 or dsRNA-GFP treated shrimp at 48 h post WSSV infection. The data is provided as the mean ± SD from triplicate assays and analyzed statistically by Student’s *t*-test (*p* < 0.01). **(D)** Survival of WSSV challenged LvTRAF3-silenced shrimp and GFP dsRNA treated shrimp. The data is provided as the mean ± SD of triplicate assays and analyzed statistically by Student’s *t*-test (^∗∗^*p* < 0.01) **(A–C)**. Experiments were performed three times with similar results and analyzed statistically by Kaplan–Meier plot (log-rank χ^2^ test) (^∗∗^*p* < 0.01) **(D)**.

To determine the function of LvTRAF3 faced with WSSV, we suppressed LvTRAF3 expression *in vivo* via the RNAi strategy. Several studies have reported that nucleic acid mimics, especially dsRNA, can strongly induce non-specific antiviral immune responses in insects, shrimp, and oyster (24). GFP was the specific gene from jellyfish, and did not exist in shrimp. To eliminate the sequence-non-specific effect of dsRNA, we selected dsRNA-GFP as the treatment for control groups, which could keep the sequence-non-specific effect without targeting any genes in shrimp. Forty-eight hours after dsRNA injection, shrimp were injected with WSSV or PBS, and their survival numbers were counted every 4 h. The silencing efficiency of *LvTRAF3* was checked by qRT-PCR at 48 h post WSSV infection. The injection of dsRNA-LvTRAF3 resulted in a significant decrease in the *LvTRAF3* transcription levels, which was down-regulated to 0.18-fold of the GFP dsRNA injection groups (control) (Figure 5A).

The expression of WSSV immediate early (IE) genes somewhat reflects viral pathogenesis (25). We chose several viral genes to investigate their expression in LvTRAF3-knockdown shrimp. We found that the expression of *wsv056*, *wsv069*, *wsv249*, and *wsv403* in the LvTRAF3-knockdown group was 7. 16-, 10. 26-, 10. 10-, and 11.35-fold higher than that in the dsRNA-GFP group, respectively (Figure 5B). These results showed that LvTRAF3 was involved in the antiviral defense against WSSV. We also found that the expression of *VP28*, a WSSV structural protein, was 10.23-fold higher in LvTRAF3-knockdown shrimp (Figure 5B). Consistent with the upregulated expression of WSSV genes, higher viral loads were observed at corresponding time points after WSSV infection. As shown in Figure 5C, the viral loads of the dsRNA-LvTRAF3 group were significantly higher than those of the control group, with a 10-fold increase being apparent. In addition, at 12 h post WSSV infection, shrimp from two groups (dsRNA-GFP and dsRNA-LvTRAF3) started to die. However, the survival rate of the dsRNA-LvTRAF3 group reached 0 post WSSV infection (108 h) faster compared with the control group (144 h). Meanwhile the survival rate of shrimp in the LvTRAF3-knockdown group was significantly lower than that in the GFP-knockdown group (χ^2^: 12.25, *p* = 0.0066 < 0.01) (Figure 5D). All of the results above indicated that LvTRAF3 played a crucial role in the innate immune defense against WSSV infection.

### LvTRAF3 Not Involved in NF-κB Mediated Immune Response

**FIGURE 6:**
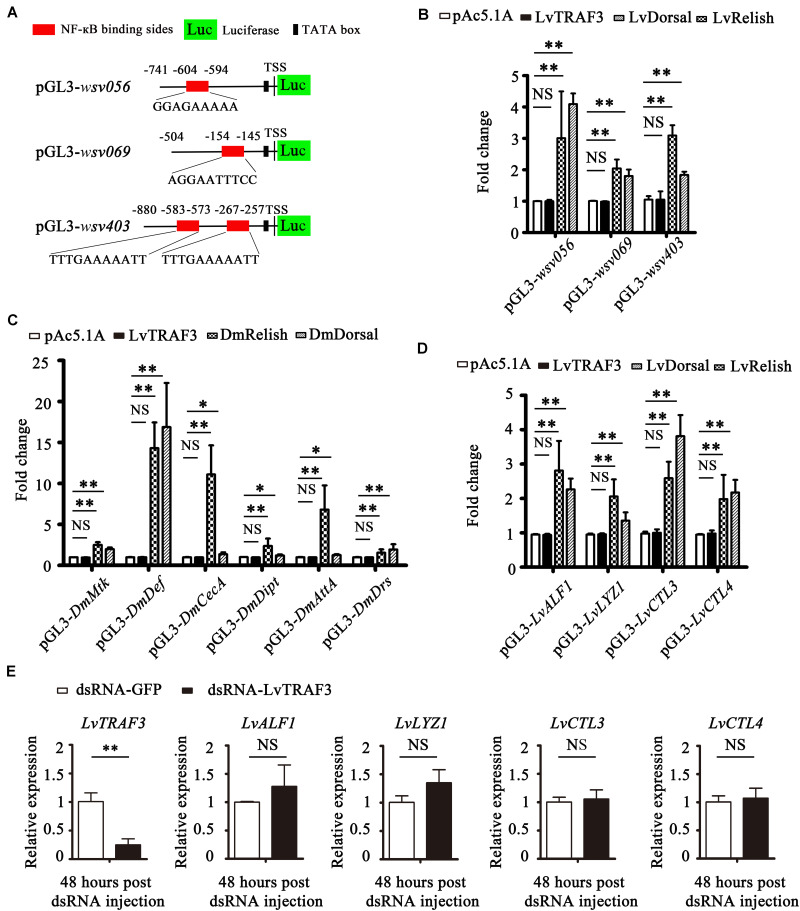
LvTRAF3 fails to regulate the expression of NF-κB pathway driven genes *in vitro* and *in vivo*. **(A)** Schematic diagram of the *wsv056*, *wsv069*, and *wsv403* promotors regions in luciferase reporter gene constructs. **(B)** Relative luciferase activities of the WSSV genes *wsv056*, *wsv069*, and *wsv403* in S2 cells. **(C)** Relative luciferase activities of *Drosophila* AMPs in S2 cells. **(D)** Relative luciferase activities of shrimp AMPs in S2 cells. **(E)** The expression of *LvTRAF3* and the shrimp immune effectors *LvALF1*, *LvLYZ1*, *LvCTL3*, and *LvCTL4* in dsRNA-LvTRAF3 or dsRNA-GFP treated shrimp at 48 h post WSSV infection. All of the data are plotted as the mean ± SD from triplicate assays and analyzed statistically by Student’s *t*-test (^∗^*p* < 0.05, ^∗∗^*p* < 0.01, NS, non significant) **(A–E)**.

It has been reported that shrimp NF-κB can induce the expression of some WSSV IE genes that contain NF-κB binding sites in their promoters, such as *wsv069* (25). In addition to *wsv069*, NF-κB binding sites were also found in the promoters of *wsv056* and *wsv403* (Figure 6A). Dual luciferase reporter assays in *Drosophila* S2 cells showed that both shrimp NF-κBs (LvDorsal and LvRelish) were able to upregulate the promoter activities of *wsv056*, *wsv069*, and *wsv403* significantly, whereas ectopic expression of LvTRAF3 had no effect on the promotor activities of *wsv056*, *wsv069*, or *wsv403* (Figure 6B).

In *Drosophila*, AMP expression is mainly regulated by NF-κB (DmDorsal, DmRelish). In details, DmRelish regulates the expression of *DmDpt* (*Diptericin*), *DmAttA* (*Attacin A*), and *DmCecA* (*Cecropin A*), while DmDorsal regulates the expression of *DmDrs* (*Drosomycin*) (26). Both DmRelish and DmDorsal can regulate the expression of both *DmMtk* (*Metchnikowin*) and *DmDef* (*Defensin*) (26). Dual luciferase reporter experiments showed that both DmDorsal and DmRelish upregulated the promoter activities of *DmMtk*, *DmDef*, *DmCecA*, *DmDipt*, *DmAttA*, and *DmDrs*, whereas over-expression of LvTRAF3 had no effect on the promoter activities of these NF-κB-mediated AMPs (Figure 6C). Likewise, *L. vannamei* NF-κB was shown to induce a lot of immune effectors, such as LvALF1 (anti-LPS-factor 1), LvCTL3 (C-type lectin 3), LvCTL4 (C-type lectin 4), and LvLYZ1 (lysozyme 1) (27–29). As shown in Figure 6D, we found that LvRelish and LvDorsal, but not LvTRAF3, could upregulate the promoter activities of *LvALF1*, *LvLYZ1*, *LvCTL3*, and *LvCTL4*. These results suggested that LvTRAF3 was not able to induce NF-κB driven *Drosophila* and shrimp AMPs *in vitro*.

To further explore whether LvTRAF3 exerted a regulatory effect on NF-κB pathway targeted genes *in vivo*, we determined the expression levels of these AMPs in LvTRAF3-silenced shrimp during WSSV infection. At 48 h post WSSV infection, dsRNA-LvTRAF3 resulted in a significant decrease of LvTRAF3 transcription levels [0.18-fold of the GFP dsRNA injection group (control)]. Yet, the mRNA levels of shrimp AMPs did not change significantly due to the knockdown of TRAF3 (Figure 6E). Together, our results revealed that LvTRAF3 could not participate in regulation of the NF-κB pathway *in vitro* or *in vivo*.

### Participation of LvTRAF3 in IRF-Vago Mediated Antiviral Responses

**FIGURE 7:**
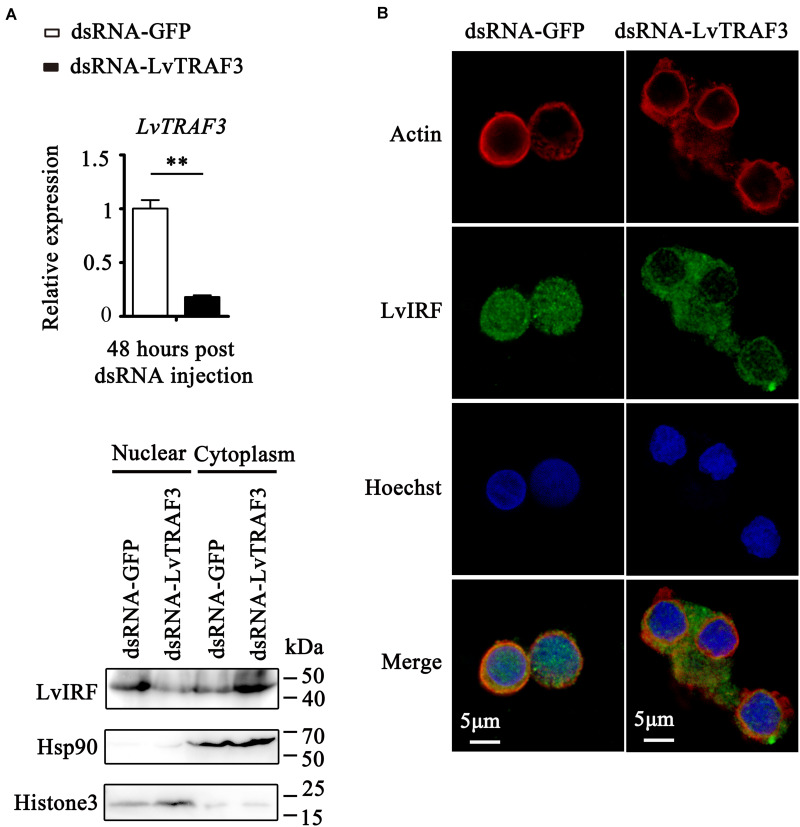
LvTRAF3 could activate LvIRF nuclear translocation. **(A)** Knockdown efficiencies of LvTRAF3 were checked by qRT-PCR (**p < 0.01). **(B)** LvIRF nuclear translocation was inhibited in LvTRAF3 silenced hemocytes. The hemocytes were collected at 48 h post dsRNA injection, and then subjected to immunofluorescence staining. β-actin was used here in order to define the shape and cytoplasmic region of cells. **(C)** The subcellular distribution of LvIRF in LvTRAF3 silenced hemocytes was detected at 48 h post dsRNA injection by western blotting. The GFP dsRNA treated hemocytes was used as a control.

In mammals, several TRAF family members can modulate IRF activity to trigger an IFN-mediated immune response (7). Interestingly, it has been reported that shrimp possess an IFN system-like antiviral pathway, as evidenced by the fact that shrimp LvIRF is able to induce the activation of LvVago4/5, invertebrate IFN-like molecules, to defend against viral infection (20). In this study, we were curious about the relationship between LvTRAF3 and the LvIRF-LvVago4/5 pathway. We firstly investigated the effect of TRAF3 on IRF nuclear translocation by immunofluorescence staining in shrimp hemocytes. The results showed that dsRNA-LvTRAF3 injection inhibited LvIRF translocated from the cytoplasm to the nucleus, while the control treatment of dsRNA-GFP did not (Figures 7A,B). To confirm the above results, we probed LvIRF translocation from the cytoplasm to the nucleus upon LvTRAF3-knockdown by using an LvIRF specific antibody. In good agreement with the results of immunofluorescence staining, we were able to detect less nuclear import of IRF in shrimp hemocytes during LvTRAF3-knockdown (Figure 7C). Our data suggested that LvTRAF3 was a key factor for mediating downstream LvIRF activation.

**FIGURE 8:**
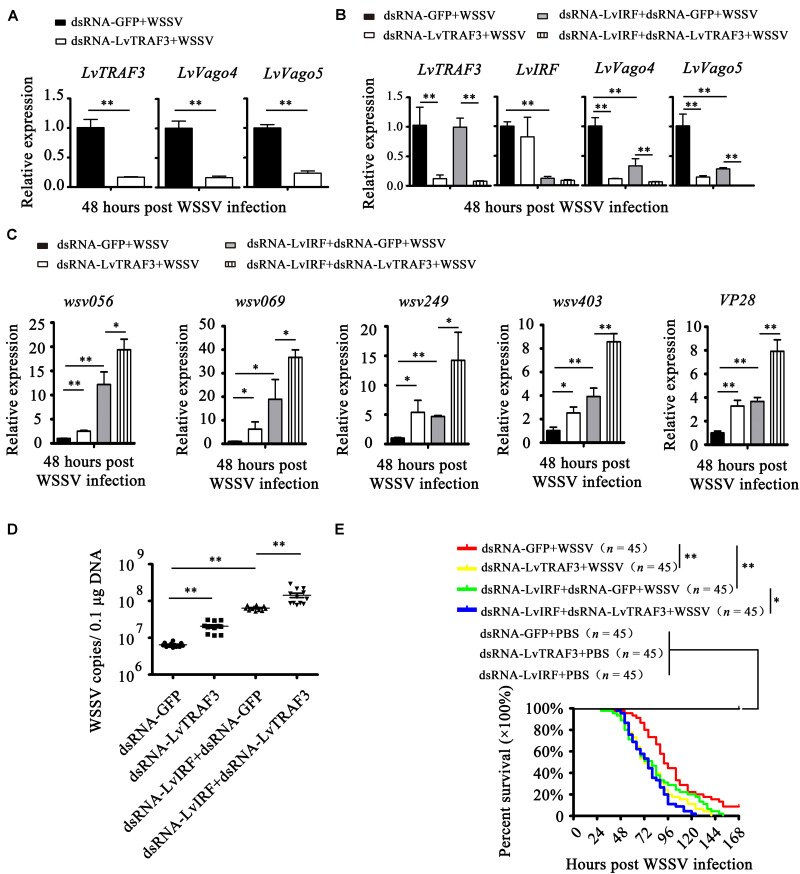
LvTRAF3 functions upstream of the LvIRF-LvVago4/5 antiviral pathway. **(A)** The expression of *LvTRAF3* and the antiviral genes *LvVago4* and *LvVago5* in dsRNA-LvTRAF3 or dsRNA-GFP treated shrimp at 48 h post WSSV infection. **(B)** The expression of *LvTRAF3* and antiviral genes *LvIRF*, *LvVago4*, and *LvVago5* in dsRNA-LvTRAF3 or dsRNA-GFP treated shrimp with or without dsRNA-LvIRF injection at 48 h post WSSV infection. **(C)** WSSV genes *wsv056*, *wsv069*, *wsv249*, *wsv403*, and *VP28* transcription in dsRNA-LvTRAF3 or dsRNA-GFP treated shrimp with or without dsRNA-LvIRF injection 48 h post WSSV infection. **(D)** WSSV copy number in dsRNA-LvTRAF3 or dsRNA-GFP treated shrimp with or without dsRNA-LvIRF injection 48 h post WSSV infection. The experiments were repeated three times with similar results. One dot represents 1 shrimp and the horizontal line represents the median of the results. The results were analyzed statistically by Student’s *t*-test (^∗∗^*p* < 0.01). **(E)** Survival of WSSV challenged dsRNA-LvTRAF3 or dsRNA-GFP treated shrimp with or without dsRNA-LvIRF injection. Experiments were performed three times with similar results and analyzed statistically by Kaplan–Meier plot (log-rank χ^2^ test) (^∗∗^*p* < 0.01, ^∗^*p* < 0.05). All of the data are plotted as the mean ± SD from triplicate assays and analyzed statistically by Student’s *t*-test (^∗^*p* < 0.05, ^∗∗^*p* < 0.01) **(A–C)**.

Then, we explored the potential function of LvTRAF3 involved in IRF-Vago pathway. As shown in Figure 8A, during WSSV infection, the suppression of LvTRAF3 by RNAi led to down-regulated expression of *LvVago4* (0.17-fold) and *LvVago5* (0.23-fold), which suggested that LvTRAF3 might be involved in the regulation of *LvVago4/5*. Next, we performed a double knockdown experiment to investigate whether LvTRAF3 regulated the expression of LvVago4/5 through upstream LvIRF. First, we observed that LvTRAF3- or LvIRF-knockdown reduced the expression of *LvVago4* and *LvVago5* during WSSV infection. qPCR assays then demonstrated that the reduction of *LvVago4* and *LvVago5* in dsRNA-LvIRF-injected shrimps became more obvious when LvTRAF3 was silenced (Figure 8B). Accordingly, dsRNA-LvTRAF3 or dsRNA-LvIRF injection led to upregulate the expressional levels of *wsv056*, *wsv069*, *wsv249*, *wsv403*, and *VP28*, while the double gene knockdown caused a more increased in the expression of those genes (Figure 8C). In agreement with this finding, enhancement of viral replication and pathogenicity by LvTRAF3 suppression was observed in LvIRF-silenced shrimp (Figures 8D,E). In detail, knockdown of LvTRAF3 caused a lower cumulative mortality in LvIRF-silenced shrimp (χ^2^: 12.42, *p* = 0.0145 < 0.05), suggesting that LvTRAF3 might defend against WSSV infection via LvIRF (Figure 8E). Taken together, these results suggested that LvTRAF3 could trigger an antiviral response via the LvIRF-LvVago4/5 pathway during WSSV infection.

## Discussion

Tumor necrosis factor receptor-associated factor family proteins play important roles in the innate immune response to viral invasion (30). In this study, we identified a TRAF3 homolog from *L. vannamei* (LvTRAF3) for the first time, explored its antiviral function and the relationship involving related immune pathways in shrimp.

In mammals, TRAFs 1 through 6 share a conserved TRAF-C terminus, which is required for the hetero- and homo-oligomerization of TRAF proteins, and recruitment to TRAF binding motifs in the cytoplasmic domains of cell surface receptors, as well as certain cytoplasmic and nuclear proteins (1). LvTRAF3 also has a TRAF-C domain at its C terminus. A sequence alignment revealed that the TRAF-C domain of LvTRAF3 was homologous to those from other species. Phylogenetic tree analysis showed that LvTRAF3 was closer to invertebrate TRAFs across evolution, suggesting that LvTRAF3 belonged to the TRAF family. Compared with mammalian TRAF2–6 and *Drosophila* TRAF6, LvTRAF3 lacks RING and zinc-finger domains, which are vital for triggering downstream kinase activity in some signaling pathways, such as the NF-κB pathway (9). Additionally, LvTRAF3 lacks coiled-coil domains, which are conserved between dTRAF3 and mammalian TRAF3 (31). The deficiency of some classical domains makes LvTRAF3 structurally different from other members, which may lead to the diversity of LvTRAF3 functions.

Studying subcellular localization can help us better understand the function of a protein. TRAF family proteins exist in the cytoplasm, which corresponds to their roles as adaptor proteins in the cytoplasm. Human TRAF4 is special in the TRAF family because it contains nuclear localization signals (NLS) and is distributed in both cytoplasm and nucleus (31). Of note, human TRAF6 lacks a NLS, and under genotoxic stress, translocation of TRAF6 from the cytosol to the nucleus facilitates the ubiquitination of p53, thus reducing apoptosis and tumorigenesis (32). Interestingly, LvTRAF3 was found to be mainly distributed in the cytoplasm with some in the nucleus, although there was no obvious NLS in the structure of this protein. We speculated that LvTRAF3 may be able to interact with some proteins that can enter the nucleus and translocated with them into the nucleus together. To our knowledge, this is the first report of the nuclear localization of an invertebrate TRAF3, and this interesting phenomenon is worthy of further exploration.

Tumor necrosis factor receptor-associated factors function as the adaptor proteins in multiple signaling pathways, and they are involved in a diversity of biological processes. It has been reported that TRAF family members from different species play crucial immunoregulatory roles in innate immune response. Faced with poly (I:C) stimuli and Sendai virus infection, *Anas platyrhynchos* TRAF6 (ApTRAF6) mediates NF-κB activation and interferon-β expression (33). In invertebrates, *Crassostrea gigas* TRAF2 (CgTRAF2) can be induced by *Vibrio alginolyticus* or Ostreid herpesvirus 1 (OsHV-1) (34), while *L. vannamei* TRAF6 (LvTRAF6) induces several kinds of AMPs during *Vibrio parahaemolyticus* or WSSV (35). In this study, we found that the expression of LvTRAF3 in the hemocyte and intestine were induced by the treatments of WSSV and poly (I:C), the conservative pathogen-associated molecular pattern (PAMP) mimics of RNA virus, which indicated that LvTRAF3 could be involved in the innate immune response for both DNA virus and RNA virus infection. Furthermore, by RNAi, LvTRAF3 was found to be crucial for shrimp to oppose WSSV infection.

Emerging studies have shown that TRAFs can signal through various pathways to stimulate the production of related effectors for defense against pathogenic invasion. For example, *Epinephelus punctatus* TRAF6 (EpTRAF6) was thought to activate an immune response via NF-κB activation, and *Danio rerio* TRAF6 (DrTRAF6) plays a protective role against pathogen invasion by inducing AMPs (36, 37). At present, the NF-κB and IRF-mediated pathways are the two well-identified ones for shrimp in response to microbial infection (12). In *L. vannamei*, NF-κB contains LvDorsal and LvRelish, which can regulate the expression of several kinds of AMPs, such as ALFs, PENs, CTLs, and LYZs, to resist diverse microbes, including WSSV (13). However, the shrimp IRF-mediated pathway, namely, the IRF-Vago-JAK/STAT axis, has been found to function similar to the invertebrate IFN system, and played vital roles in the defense against viral (WSSV) infection (20). How, unfortunately, the invertebrate TRAFs, including LvTRAF3, mediated signaling pathways are largely unknown. In our study, we found that knockdown of LvTRAF3 had no effect on the expression of NF-κB-mediated AMPs, which indicated that LvTRAF3 was not able to signal through the NF-κB pathway. This result was consistent with LvTRAF3 protein structure, which lacks a conserved RING domain or a Zinc finger domain. In previous reports, the RING domain and Zinc finger domain were found to be required for the activation of the NF-κB pathway (9). Instead, we discovered that LvTRAF3 could activate LvIRF translocation, and trigger the shrimp IRF-Vago pathway in response to WSSV infection. Interestingly, in insects, the recognition of viral dsRNA by *Drosophila* Dicer2 can induce Vago activation, and *Culex* TRAF-Rel2 signaling pathway is involved in the activation of Vago (38). Nevertheless, the definite molecular mechanism by which LvTRAF3 modulated shrimp IRF-Vago pathways remains unknown and requires for further investigation.

Collectively, we identified the TRAF3 homolog from *L. vannamei* and explored its function during WSSV infection. Our results demonstrated that LvTRAF3 responded to WSSV infection and altered resistance to viral infection. In addition, we found that LvTRAF3 defended against WSSV infection via mediating the activation of the IRF-Vago pathway but not the NF-κB pathway.

## Data Availability Statement

The datasets presented in this study can be found in online repositories. The names of the repository/repositories and accession number(s) can be found in the article/Supplementary Material.

## Author Contributions

CL conceived and designed the experiments. HL, QF, SW, RC, XJ, and PZ performed the experiments and analyzed the data. HL wrote the draft manuscript. JH, HL, and CL acquired the funding. CL was responsible for forming the hypothesis, project development, data coordination, and writing, finalizing, and submitting the manuscript. All authors discussed the results and approved the final version.

## Conflict of Interest

RC and XJ where employed by Guangdong Hisenor Group Co., Ltd. The remaining authors declare that the research was conducted in the absence of any commercial or financial relationships that could be construed as a potential conflict of interest.

## Footnotes

1 http://smart.embl-heidelberg.de/

2 http://www.ncbi.nlm.nih.gov/

3 http://www.nrbsc.org/gfx/genedoc/

## Supplementary Material

TABLE S1 5′ flanking regions of reporter gene plasmids, including WSSV genes, *D. melanogaster* and *L. vannamei* antimicrobial peptides.
