# Supplementary material for: TNF-Receptor-Associated Factor 3 in Litopenaeus vannamei Restricts White Spot Syndrome Virus Infection Through the IRF-Vago Antiviral Pathway
Source: Front Immunol. 2020 Sep 11;11:2110. doi: 10.3389/fimmu.2020.02110 (PMC7518466; doi:10.3389/fimmu.2020.02110)
Supplement: TABLE S1 — 5′ flanking regions of reporter gene plasmids, including WSSV genes, D. melanogaster and L. vannamei antimicrobial peptides. [file Table_1.DOC]

**5' flanking regions of reporter gene plasmids including WSSV genes, *Drosophila* *melanogaster* and *Litopenaeus vannamei* antimicrobial peptides. GenBank accession numbers are showed and the location of primers are underlined.**

**>wsv056 NC_003225.1**

CTGGAGCAAATTTCTGGAAGGAGTTTGGGGCCATTGTTAGGCTATGCGGAGCAACACAAGAGTGTATTCGGCAGTGATGGTGGATGAAATGTGTGCAAAATATGCTTAGTGGTTAGCTTATATAGTCTGCGTCAGTGGGAGAAAAAATGTCCCAGAAACTAGTTGGGATGTTTCTGGGCTCTAGCCACTCTAGAGATGTCTGGAGCGCACCCTAGCACCCTCTTTTTCCCCCTTCATCTTCATCTCAAAAACTTTTCAAAAATTTTTCTGGGTCACTCCAGTTTAGGGGGTGGACCGCTGAGTCGATCGAATGTCAAGTTCCGAGGGTGGACCGCTGGGTCGGCCCAATGTCAGATTACACGAAGGGGCTAGAAATGTATACCAGAAATAATGCACAGAAATCTTTAGAATCATTTCTGGAGCAAGGCAGAACTGCCTGTATTCACTAACCCTTTTCCCCCTTCATCTTCATCTCAAAAACTTTTCAAAAATTTTTCTGGGTCACTCCAGTTTAGGGGATGGACCGCTGAGTCGATCGAATGTCAAGTTCGAAGGGTGGACCGCTGGGTCGACCCAATGTCAGATTACACGAAGGGGCTAGAAATGTATAACAGAAATAATGTACCAGATGTGAGTCAAACCGTTTCTGGGTTCTAGCCGACCCCGGTATGAAAGATGTGGGAGAATTTTTGGGGGGGGGGGTGTTAGTCTATATATAAGACTGTGAGCTCCTTGCTAGTA

**wsv056-F：GGGGTACCCTGGAGCAAATTTCTGGAAGGAGTT**

**wsv056-R：GGAGATCTTACTAGCAAGGAGCTCACAGTCTTAT**

**>wsv069 NC_003225.1**

ATGTGGCTAATGGAGAATTGTCGTGCCAGTTGTATCAGCGAAGCGGAGATGTCGGGTTGGGCGTGCCCTTCAATATTGCATCATACTCTCTTCTGACTCATCTGATGGCCAGTATGGTGGGTCTAAAACCGGGAGAGTTTATCCTCACTCTTGGTGACGCACACATTTATAATACCCACATTGAGGTGTTAAAGAAGCAGTTGTGCCGCGTCCCTAGACCATTCCCTAAGTTGAGGATTTTAATGGCTCCAGAAAAAATTGAGGACTTTACTATCGACATGTTTTATCTTGAGGGGTATCAACCACACAGTGGAAACTTGCAGATGAAAATGGCTGTTTGAATCATGTTAAGGAATTTCCTTGTTACTCATTTATTCCTAGAAATGGTGTAATCGCTGTTGTGGGCGGAGCATATTTGTGTATATAAGAGCCCGTGTTAGCTCCTCGATTCAGTCACAAGAGCGCACACACACGCTTATAACTAGCTCTCTCTCTCCACTCAAG

**wsv069-F：GGGGTACCATGTGGCTAATGGAGAATTG**

**wsv069-R：GGAGATCTCTTGAGTGGAGAGAGAGAGC**

**>wsv403 NC_003225.1**

AGTACTCCGTAGCCAACATATACACATGAACACATGAGGCGGTCTACAACAGAAAGAGAACTGATAGCTGTTCCAGATATCTGGGTCGGCCAGAACCCAGAAACGTTTCAACTCATTTCTGGACAAGCCATTTCTGGAAAGGGGTACAATTTCTTATAACTGGTATATCATTTCTGGTATAATTTCTGGCACCTTCGTGCAATCTGACATTGGGTCGACCCAGCGATCCACCCTCCGAACTTGACATCGGGCCTACCTAGCGGTCCACCCTCTAAACTCGAGTGAGCTGAAAAAATTTTTGAAAAATTTTTGATGAAGGAATATAGTAGTATAGTGCCCGCCAAAGCATACACACACTCGCCCATGCTCGTCTAGCTGATAGCTGTTCCAGATATCTGGGTCGGCCAGAACCCAGAAACGTTTCAACTCATTTCTGGACAAGTCATTTCTGGAAAGGGGGTACAATTTCTTATAACTGGTATATTATTTCTGGTATAATTTCAGGCACCTTCGTGCAATCTGACATTGGGTCGACCCAGCGGTCCACCCTCCGAACTTGACATCGGGCCTACCCAGCGGTCCACCCTCTAAACTCGAGTGACGCAGAAAAATTTTTGAAAAATTTTTGATGAAGAGATTGAGTAAAATTTCTTGACGATAAGAGGAGGCAGTAGGTGAGGCTGCTTGTTTGATGTGTCAGCCACATCTGCGTCATACATTATATTTCCAAGAATTTTGCTGACGTCAATGGACCATAAAAGGCTTTGTACGTCCAGAGACAAGTTTTAGTCTGATAGATTTCTTAAAAAAAAGAGGGTGGGAGGTTTGTTTTTGTGGGTTCTGTGTGTGTATAAAAGATAGGTGCAAAGGTAGAGAATCATCATATGGACAAAATCTGTCCATGAGACCTCAGTAGAGAACGCACCATGGTTGCTTCAACTCCGTGTCCAGGCCCAGGACCAGTTCCAACCCAAGAACTTCTTTCTACAAACTTTCTTGAAGCTCACAAGCTTGTCGTGGAACTTCTTCTCCCGTCCTACAGTAGTGATGTAGTTTATTGTGACTCTGAGACGTAC

**wsv403-F：GGGGTACCAGTACTCCGTAGCCAACATATACAC**

**wsv403-R：GGAGATCTGTACGTCTCAGAGTCACAATAAACTAC**

**>*****Drosophila melanogaster* Cecropins A (CecA) AAF57025.1**

GAAAAACAACTAAGTTACTAACGCAAGACTTTTAGTTAAGTTAGTTAATATAGACCGAGATGTATGTACATACATACCGCTTTCGCTTACAATAAAATGTTAAATAAGTTTTCAGATTCGTACGTGCTCAGTAAACAATTATTTTTTATTGTCATTTAATGCCTATTGAATTTTTCAAACTTAATTTAGTGCCTTTAGTAAAATATTGTAGTGATTCCCCTCGAAAAATACCACAAATTGGATGCGTTTATGTAAATAAATTGCCCTTGAGTGATAGAGTAAATTTGAATTTGACTGTCTTAGAAAGATAGAAAGAGATCAATTCAAAATGCCAAAAGGATAGAGTTATTAAAGCTCTAATTCAAATTGGCCCAGAACCGTTTAAAGGATATTACAATTTGTAATTTACATATTTGGATTATAGCATTGAAATCCCCGATTGTTCCCTAGATGTGCAGATGTGTGCTTGGAATCAGATCGGTTACCTTCAGTGTACTTTTCTCTGCAAAAATCCCCGTGCATGCCTTATCTGTCATTTTGTTTTTCAAGCTGGCTGTTCGCCTATAAAAGCTCTCGCCTTTTGTATCGCAGTCATCAGTCGCTCAGACCTCACTGCAATATCAATATCTTTAGCTTCTCCTAAGAAAAAATCAAGAAAATATCACC

**DmCecA-F: GGGGTACCGAAAAACAACTAAGTTACTAACGCA**

**DmCecA-R: GGAAGATCTGGTGATATTTTCTTGATTTTTTCTT**

**>*Drosophila melanogaster* Metchnikowin (Mtk) AAF58139.1**

ATTTAAAGGGTAGCGCCACGTTCAACCTCTTTTGCAGCCCCATTCTGCTGCGAGAAAACTAACAAAGTGCTCTAATCGAGCCAAGGGGCAATTTCTTGTGTTGCTGCAGCTGCACTTTGCACCTCCGCATCCGTGCACCCAAAAACCCGCTTTCTAGATGTTCTCATCATGCACTGAAAAAGAATCCAAATTTTTACAAGAAATAGTTTAAAATTAGGTAATGTGAAAGATATCGGCACACGGACAGGCCGAATTATCTGTTGTAAAACTAGCTGCTCAGTTATTAAAAACATTTGTAGTTGCTGACGTTTCCATACAGAGACTAATTTTATTTTCACGGACAGGGGTTTTCCGCTTTAATTGCTTCATTTTTGTTGCTTTATTGCGTGTATATTGCCCCACAAAACAGATATAAATCATTCGCGCATATCGTAAATGTTGGTAGAAAATGACAAACAAGAGAAAAAAGATGATTAAAAGCTTCAAGACAATCCTCTATAGGATCTGATTAAATATGAATATTTTATTTATTAGTTTTCTTTCTGTGTACGGCTTAGAAGGCAGAAGCTGCGAGGGGCGTAGGGCAGTGGGCGTGGCTCCGTGTTGACGCATGTTGACTATGCCTTTGAATGGCTGCCGTGGTTGTCGGTGGGTAATTTGCAATGCAGAAAAACCAACAGGGCGCTAAAAAGGAGAGTGTTTTCGTGGGAGGTGGAGATGGTCACTGGGGGCAACATAAATATTCAGCGAGAAACGTCATATTTACATTTAGTCTAGGCTGATAATCCGGGACCGTGGGAAGTCCCCTTTGGGTGGTGCTGGCTGGGTTCCCCTGGCCACAATCGGTTATCTGCCCCCGGCTGACACTTGCCCGTCATTCATTCGGCTGCTTATCGCAGAAGCTCAAATAAAAAGTCCCCAATCTGCGACTCGTTTGTCTGGGACTGAGCTATAAAAGCCTCACCATCTCAACGCTCAAAGCATCAATCAATTCCCGCCACCGAGCTAAG

**DmMtk-F: GGGGTACCATTTAAAGGGTAGCGCCACGTTC**

**DmMtk-R: GGAAGATCTCTTAGCTCGGTGGCGGGAATTGATT**

**>*Drosophila melanogaster* Defensin (Def) AAF58855.1**

TGACGCCAAAATGCAAGACAAGACAACCTGGTCGATAACAAAGGTAAACAGGCAACGGCCAGCCAAGGAGCAGGGCAACTGAAAAAGCCTCGGTCGTGGGATTCGATCGGGATTGTCGGCTCAGCGCGACTGGGCTGAGAACCAGATGCAGATGCCGATACAGATATAGATACACGTACGCGCAGATACGGATTCAGATACAAGTACACCCGCCCCTGCCGCTGTATGCCCAACTAATCATTGTGTGATTCTTGTTTGTTTATTTGCCCGGCATTATGAAGAGACTTTTCGGTAGAAATTATTTATTGTCGCATGTGTTTATGTATCCGTAACCGAGTATCTCAGTTGCTTGAGCCAACTGTGTAGCTGTGTAGCTGTGAGTATAGCCCTTAAAGTGGCACCCAATCGGTCAGTTAGCTAGAAATTCAGATGATTAAATATGGATTCCCCTACATCAGCTAATTTCAACAGTTTGGGAGTAATAAAATCGAAATTGGATGCTACTAAAGGGCACATATTTACTTAGGCTTTTATCAACGTTGCATATATACAAATATCCTGCATATTTCGCAAACCAAAGATTCTTTCTCAAGTAAGGCCTAAACAATTTGAAATGGTTAATTTCGTAGATGTTGCTTTTTACAATTAACTTGTCATGTGGAATATACTTTACTGCCTAAAATTTAAGGCAGTTAAAATCCCTAGAAATGCAAATAACTTATTGCAGAAACGGGCTCTGTCGGCTGTATTTTGCTCTTATCTATGAAATATTGTCAATATTTTCCAGGCAAAGCACATGAAATAATGATCTAGACAACGGTTTCTCCCATTTGCAGTGAACTTAAAAATTAAAAACCCCCGAGACGTGTCTTCCTGCACAGAAAAAGAGACAATGGGAAGGTAAGTCACCGGGTGGGAGTCCCTGGGCCGAATCGATCAGCCCGTCGCATTGCTATATAAGCTCGGCGAAACCACAATCTGCAACAACAGTATCTCTCCAGTTGTATTCCAAG

**DmDef-F: GGGGTACCTGACGCCAAAATGCAAGACAAG**

**DmDef-R: GGAAGATCTCTTGGAATACAACTGGAGAGATACT**

**>*Drosophila melanogaster* Attacin A (AttA)** [**AY056895.1**](http://www.ncbi.nlm.nih.gov/nucleotide/16555203?report=genbank&log$=nuclalign&blast_rank=4&RID=6BB4RKDM014)

GCCATCAGGCCACCACCCATTCTGCCCGCCTAAAGATGTGTGCATACCGCGGAGAAGTCATCCGATCAAATTTGTTTTGAAAAATCTTTATAAAAATTGTGAATTTTTTACTTTCTGCAAACAGTAAGCAATAAACACACGAAAGACAGCAATTAATAATCTTCAATCAATTGTGACACAATGAGGGGTTCCCATCGCTTATCAGCGGTTTTTGTACCGAATCTGCTGAGCTCTAGAGCTGATAAGAAATATACTTGCTCAAAACAAAACCACAAAAGTCACGTTTGAGAGAAAAAAAGCCTAAACGAATTTAATTCGCGACTCATATGAATCACAAACCTGTTCTATAGCACGTTCTTCTTAAATTCTAGCGAAATAACCAGATGGCTGCAAATCATAATGAATGGGTTTGTCCCCTAAAAAAAACGAACTGACAAGCCCCCTTATAAAACTTATTTATTAATAGATTAGTTCGTATATAATTGCATATGTAAATACTTTAAATAGAACAAATTATTACGACATTTAAAAATATATATCCTGGTTTTTAAAAACAGGGTTTGAAAAAAATGTTTATAAGCTAATTACCTGGTAGATTATATTTTTCAGTGCAAACTTTTCGTTGACACTGCGGGTTAAAGTTTCCGCTCTCCTCTCTTTCGGCTCGCATCCTTTTTGCCCGCTCCTGCGCAGAAAATTCAATTAGGATTCGGCTGAAACTTCACTCAAATCCTGCCGCTCTCACCGTCGCTCTTTATTTCGCTCGCCTTCCCTTTCCGGCTCCCCCAGAATAATCCCTCCACGCAAAATAACGTATTGATAAAGCCTGACATCAAGTGAGAAATACGATAGAGAATCCCTTATAACTGTCAAATGCCGACCTGCGCAAGATGAGGATGCACTCCTCCATCAAGACACAAAAGAAACCACTTGGAGACGCTGACAGAGGTTCTGCGGCGAGGGTGAAACTGGACAATGCAGCAACAAGTGGCGTCAATGGGTCGCAAAAAAGGGGAGGTGATGAGGTCAAGTCGCACAGCCACACAAGCAAACAGCAGAAGTAAACCACCATCACATCTGAGCGGGGAATTTCGCTTTGATAAGGCATCCAGGCCGAGATCGGCAATCAGATGAATCATGTCAATCATCAGAAAAGTTCTTCCCCGCATCTTGAGGTATAAAACCGATGCATTGGACACCTTGAAACATCAGTCAGCTCCAGCAATCCAGTTCAGCAAC

**DmAttA-F: GGGGTACCGCCATCAGGCCACCACCCATTCTG**

**DmAttA-R: GGAAGATCTGTTGCTGAACTGGATTGCTGGAGCTGAC**

**>*Drosophila melanogaster* Drosomycin (Drs)** [**AJ885064.1**](http://www.ncbi.nlm.nih.gov/nucleotide/83998954?report=genbank&log$=nucltop&blast_rank=4&RID=6BB9T2E1015)

CAATGAAAGTGATAATACGAATTGACCATGTAGCAATTTGTTTTGTGTCTATAGACTGAATTTTTTCCCCTACATTAATCAAAATTATATTTTTATATTTTATTTTGAGTTTACTTGGGTTTTTCATGAAATTAAAGATTAACCTGGGGTTTTTACAATCCATTACGATAGGCTTTCTGTTCATTGCATCTATAGCCTCTGTACTTTTCCGTGCATTCTTAAAGCAAAAGCATACATGTATATCTTCAATTCAAGTATATCTGCAATTTAGTTTGCTTATCTGGAGTCGCTGTATCCCGACCTATCCAAACTCGCGTCCCAGTCAAAGGTAAACCATTTTTATTTAGTTCCCAGCCTCTGGTATTTGTTGTTATTTATCGGTGACTTTTTGAAATTTATTCAATTAATTTCAGTCTTTGTGCTCTTGATAACACGATTTCCTCGTTATTCATTTAGTTTGGGTTTAACCAAAACCCTTTAGCGAATCATTTTTGCTGGACAGTCCAGTTGAATTCGGTATTCTACACACAAAGCTCATCTTACAGTGAAAAGTGTTACTTTATGAAATACAAATGAGGCTCTAAGCAATGCTTTTCGCTTACGCTTTTCGATAAGCGTACAAGTAGTTCCCCTACCGAAGGCCTATAAATGTGACTGCACATGTATCATCATAATTTGTTGATATACTTCGTTTATACCCGACTACGCATCGGCTAAAGCTGAGGGATCGGTGCACTATATAAGCTTCTCCTCGAAGTTCCCAAGCCACAAGTCGCTGATAATTCAAACAGAAATCATTTACCAAGCTCCGTGAGAACCTTTTCCAAT

**DmDrs-F: GGGGTACCCAATGAAAGTGATAATACGAATTGACC**

**DmDrs-R: GGAAGATCTATTGGAAAAGGTTCTCACGGAG**

**>*Drosophila melanogaster* diptericin (Dipt) AF019020.1**

TGCTATGGTCAGTTAGTACACGTTTTGCCAGCTAATGTTTATATTTTTCCCTCAGTAAATTATCCATTTGATCACCAGAGACGTCAGTTCAAATATCACACTTTTGCCGGAACTATTGACAAAGATGTTGTTATGGGAATCACTGTCGTAATGCTATGTCAGATAGTATCTTCGATTCTACTATGCTTTGCATTAGATGAGGTGAAGTTTAAGAATATAAAATCCTGGCGGTATATTAATTAGTTTTCATTTTAGAAGAAAAGAACATGTCTTATTTACGGAATTTTCATTAGCCTAACTTTTCCGTGTGTCTGTCTCCCAGTTTGGCCATTAGCTGGTAAGTTTTTAGAATTTTCAAAAATCTTTTTCGAGGCATCTAATAGTGTGTTTCAACAGTAACACATGTCAGTCTTGTTTTGGTAGTGCTGAGTTATTACTTTGAGTAGTTTAGAAAATGTTGTAAAAAATTGTAATTATTTTGGTCCCTGTATACAAAATATGTTTGGAGGTTCATTCATTTAAAAATGGGAATTATGGGTATTGATATAGAATATATTTATAAAGTAGTAAGATATAATTTAATACATGAGTAGAAAGAGTTTACTTTTATATAAAGCCAGTTTTGACAAACAATAGGTGGTTTATATATTATAACTGACTAAATTAAATTAAGACTAAGTCATACAAGAGTAAGTTATTTTTCTATGGGCCCAAAAGTATAGTATAATTTTGGTTATAACAAGTAACTTTACTGATAAGACTTGGATTCTCTTTATAATATATTTAACAGAGATGTATATGATGCAATTTATAGAATTTAAAAAATCTTAAGAAACTTAAAATGTTGCTTCAAGATCCTGGTCATCATTGCCCCAGGGAAATTCCGTCTTTTCCGGTGGACCTTCACCCCTTGAACCAATTTCAAGCTCTATATAATCGGCAGATGACTGGGCTGTGACGTGTCCCAGCTGTAAAGTAAATATGCATATATATTTTTTTTTATATTTTTTTATTTTCGGATTAATTGTATATTTTTCCTTTGCCGCACTCACCCAGCAGACCAAAAAAACGGCCAATATTTTCATTAATATCGATTGTAACAACATTTCTCTCGGCTGCCTCGGCACTCAACTGCTGATGGGAAACTGTTTTTACGTTTGCTCAACCTCTGCTTTTAATCAATTATCACTTATTAAATATTTATATTTGTTTTTTTTTTTGTTTGCCTGCATACAAACATACATCGCTCTTTGTCTGTCGCCGCGGAGAGGTTTTAAAATTAATCCGTGGAACTGGGAAAAGGATGAACTTTCGTTTATTTTCGAAGGGAAATCATTAATGTTTTAATTGTTAATAATAAGCTGGGAGGTTGGGATATTGTTCTTAAGATACATATTTAAAAACTTCGTGGAATAAGAGGTTACAAATTTTATCATTTAATAAGTATTTAACCTCTTGTTTGTCAAATGAAAATAAGGTGTGAGTCCTCGTTTAAGAAAGATCCCCTGGTGGTATTTGTTTTTGCATCGGGGATTCCTTTTTTATGACCGGTAATCAATCTTGGGTTCTAATTATGAGACAATAACCGCCGTAGGTATACTTTCTGAGTAGATAAGGTGACATCGGGGATTCCTTTTGGAAAGCGGCCTATAAAAGAGCATCGAAACTGCAGCAAAGGTATCAGTCAGCATATTCCAGTTCTTCAATTGAGAACAACTGAGATGCAGTTCACCATTGCCGTCGCCTTACTTTGCTGCGCAATCGCTTCTACTTTG

**DmDipt-F: TTGGTACCTGCTATGGTCAGTTAGTACACG**

**DmDipt-R: GGAGATCTCAAAGTAGAAGCGATTGCGCAGC**

**>*Litopeneaus vannamei* ALF1 EW713395**

CTTGATTAGCCGATCCCAGACATCCTGCAGCCCAACAACCAAAGCCAGGCACGACGGGCGTGCGGGAGGACAGCCCTGCCTTCAGGATAAGCTCCCGGCGAGGCATGAGCGCGGAAATGCAGTCCTTATGCGCAGTCGGCGACGGACAGGCTTCCGAGCAACACCGCTTCCGCATTCGGCCTTGACTTCGGGGGGGAAAACACGACGATGCGGGTGCTGGTCAGCTCTGTAGT

**LvALF1-F: GGGGTACCCTTGATTAGCCGATCCCAGAC**

**LvALF1-R: GGAGATCTACTACAGAGCTGACCAGCACCC**

**>*Litopeneaus vannamei* LYZ1 JN039375.1**

CTATGGTGAATGCCACCGGGCAGTGCAGACGCTTTAAATCCTTCCTCTGATTCAATAACAAGACGTTATTCAATCTGAGTAGTTCTTACTCCATACAACCAGAATGCTTCCCACGATTTCAATTAATGATAGCCTAGAATAGTATATTGCAAAATGTCATATTCTATACCTTGGGGAAGCATTCCTTTACTCAAAGTACAGCGTGATAGTTAATTCTTTGAATACTAATGACGTTTCTTGTTCTATTTAATAACTCGTATTTGTGTATTTGTGATGTGACAGCCGAATGGCCTTAACCAACTGACTACGGCGAATTGTTAACAAAAAAAGGCGACTAATCCCTGCATGGCGGGCGGGTGCTATTACATTGACAAAGCTTCTGCGCGAAGTCGGTAGGCTTGCCAACGTAAGGTAAAGGGATTATATCTTCAGGGTAAATTTTGTGCTCACCGATGTGTCATCTCAAAGAGTAACAAAATTATTGTCTAATCAAATGAATGACAACTCTCCAGAAGGTTTAAAATTCGTGTTTATGATCTGATCATGCTCTTGTTTGTAGGACAAAAGTAAATTGATTTGCATTAATCCCACATTACTTTTTTCCTTGTACTAAATAATATTTCGAAATACATCAGAGATACTCCTGATTATACAAAGGTGCAACTTTTTGTCATCTGTTTCACATCGCCCTTGTTATTTGTCAATCAGGTTGCATTCGGTACTAAAATGATAAGTTCATACTGCGTAGTCAAAATGCATATCAAAAGTGATAAACTCCGATAAACATGGGTGAATTGGCTCTGATAGAGGAAAGGCCAACTGCTGCTCTATGCATTATCTCACGCTCCTCAAAACAGCTTTCGGGTAAGCGGACAACTGACGTCACGTGGGAAAACAATAATTCTTGTAGATGTATAAATAACATAATCAGCAAGGAAGGGCCGCAGACACAGCCAAGCAACTTACACTTCGGAACC

**LvLyz1-F：GGGGTACCCTATGGTGAATGCCACCGGGCAG**

**LvLyz1-R：GGAGATCTGGTTCCGAAGTGTAAGTTGCTTG**

**>*Litopenaeus vannamei* CTL3 AGV68681.1**

GTGTTATTTTGTATAATGCATTCACACATGCATATCTGAAACAAAATTTCTTTTCCTCTTTCCCAACAAGGCACAGGCTCCTAACAAAAAGGAATTTCCTCCGCCAGCTGTCATCTGTCTCGATCTTCCCATCTCAGGGTAACACATAACAACTCGCTGGCCAATATAAAGCACAGCATTGCTCTTTTGAAGGCAATACAAACGCACAAAATGATGTTCT

**LvCTL3-F：GGGGTACCGTGTTATTTTGTATAATGCATTCACAC**

**LvCTL3-R：GGCTCGAGAGAACATCATTTTGTGCGTTTGTAT**

**>*Litopenaeus vannamei* CTL4 KM387560**

GAAGCGACAGGCTCTCTCCAAGTGTGGGAAAAATCTCTTATTAGACAGAAGATACCGTACGTGTATATAAAGGAGCCCGTCGGTCACGTTCCCATTCGCCTCTGAAA

**LvCTL4-F: GGGGTACCGAAGCGACAGGCTCTCTCCA**

**LvCTL4-R: GGCTCGAGTTTCAGAGGCGAATGGGAAC**
